# Tuberculosis and sarcoidosis: The continuing enigma

**DOI:** 10.4103/0970-2113.45194

**Published:** 2009

**Authors:** Dheeraj Gupta

**Affiliations:** *Postgraduate Institute of Medical Education and Research, Chandigarh - 160 012, India. E-mail: dheeraj@indiachest.org*

Though the famous Norwegian dermatologist Caesar Boeck who probably for the first time described sarcoidosis as “multiple benign sarcoid of the skin” in 1899, thinking that the histological features resembled sarcoma, its remarkable similarity to tuberculosis in both clinical and histological features was soon noticed and a possible link between the two conditions has been debated ever since.^1^ The possible relationship can have implications in three areas: a) pathogenesis, b) diagnosis, and c) treatment.

The granulomatous inflammation in sarcoidosis is believed to be in response to the continued presentation of a poorly degradable, and yet to be identified antigen.^2^ Numerous etiologic agents have been incriminated, both infective and noninfective.^3^ Among the infective agents, the two strong contenders are the propionibacterium and the mycobacterium.^4^ However recently, *Propionibacterium acnes* was shown to be a common commensal on culture and polymerase chain reaction (PCR) analysis from lung tissues and lymph nodes of subjects with and without sarcoidosis.^5^ Also, the inability to isolate mycobacteria by histological staining or culture from tissues in sarcoidosis continues to be one of the strongest arguments against a potential role of mycobacteria. Of late, molecular analysis (such as PCR techniques) for nucleic acids of the *Mycobaterium tuberculosis* (MTB) or non-tubercular mycobateria (NTM) have added more insight into the issue. A recent meta-analysis by us of all such studies conducted from 1981-2006 revealed 31 studies that had used PCR for nucleic acid amplification followed by identification of nucleic acid sequences specific for different types of mycobacteria.^6^ Overall, 231 out of the 874 patients were positive for mycobacteria with a positive signal rate of 26.4% (95% CI, 23.6–29.5), and the odds of finding mycobacteria in samples of patients with sarcoidosis versus controls were 9.67 (95% CI, 4.56–20.5).^6^ These results clearly show that the association does exist in a significant proportion of patients.^7^ Moreover, all these studies have been from populations with low prevalence (exposure) of tuberculosis and the possible role of tuberculosis in causation of sarcoidosis in high prevalence countries including India remains to be explored.

Tuberculosis, as a differential diagnosis of sarcoidosis poses even a greater challenge to clinicians, particularly in countries like India with a high prevalence of tuberculosis. The tuberculin sensitivity is depressed in sarcoidosis even in the background of high prevalence of tuberculosis and a cut-off of 10 mm reaction to 5TU tuberculin test has virtually 100% sensitivity for sarcoidosis, however, it is not specific.^8^ The problem is further confounded by the coexistence of two diseases.^9^ The diagnosis of sarcoidosis rests on a constellation of clinical, radiological, histopathological, and laboratory data.^10^

Finally, if indeed tuberculosis is a causal factor in sarcoidosis, then the hypothesis can be further reinforced, if antitubercular therapy (ATT) is useful in treatment of sarcoidosis. Very few trials have been conducted in the past but the results of these trials have been discouraging. These trials were generally small studies and limited by time bias and used older regimens based on isoniazid, amino-salicylic acid, and streptomycin.^11^–^12^ In our experience, nearly one-third of patients who are finally diagnosed to have sarcoidosis, have received ATT for variable lengths of time, but its impact on the final outcome of sarcoidosis has not been studied. Role of antimicrobial therapy in sarcoidosis has recently been reviewed and it gives us a definite thought for the future.^13^
